# A machine learning approach in the non-invasive prediction of intracranial pressure using Modified Photoplethysmography

**DOI:** 10.1371/journal.pone.0275417

**Published:** 2022-09-29

**Authors:** Anmar Abdul-Rahman, William Morgan, Dao-Yi Yu

**Affiliations:** 1 Department of Ophthalmology, Counties Manukau District Health Board, Auckland, New Zealand; 2 Centre for Ophthalmology and Visual Science, The University of Western Australia, Perth, Australia; 3 Lions Eye Institute, University of Western Australia, Perth, Australia; Sant Longowal Institute of Engineering and Technology, INDIA

## Abstract

The ideal Intracranial pressure (ICP) estimation method should be accurate, reliable, cost-effective, compact, and associated with minimal morbidity/mortality. To this end several described non-invasive methods in ICP estimation have yielded promising results, however the reliability of these techniques have yet to supersede invasive methods of ICP measurement. Over several publications, we described a novel imaging method of Modified Photoplethysmography in the evaluation of the retinal vascular pulse parameters decomposed in the Fourier domain, which enables computationally efficient information filtering of the retinal vascular pulse wave. We applied this method in a population of 21 subjects undergoing lumbar puncture manometry. A regression model was derived by applying an Extreme Gradient Boost (XGB) machine learning algorithm using retinal vascular pulse harmonic regression waveform amplitude (HRW_a_), first and second harmonic cosine and sine coefficients (a_n1,2_, b_n1,2_) among other features. Gain and SHapley Additive exPlanation (SHAP) values ranked feature importance in the model. Agreement between the predicted ICP mean, median and peak density with measured ICP was assessed using Bland-Altman bias±standard error. Feature gain of intraocular pressure (IOP_i_) (arterial = 0.6092, venous = 0.5476), and of the Fourier coefficients, a_n1_ (arterial = 0.1000, venous = 0.1024) ranked highest in the XGB model for both vascular systems. The arterial model SHAP values demonstrated the importance of the laterality of the tested eye (1.2477), which was less prominent in the venous model (0.8710). External validation was achieved using seven hold-out test cases, where the median venous predicted ICP showed better agreement with measured ICP. Although the Bland-Altman bias from the venous model (0.034±1.8013 cm water (p<0.99)) was lower compared to that of the arterial model (0.139±1.6545 cm water (p<0.94)), the arterial model provided a potential avenue for internal validation of the prediction. This approach can potentially be integrated into a neurological clinical decision algorithm to evaluate the indication for lumbar puncture.

## Introduction

Investigation of the physiological properties of the human cerebrospinal fluid was first described in 1891 when Quinke published his studies on the diagnostic and therapeutic applications of lumbar puncture. He standardized the technique of intracranial pressure (ICP) measurement by connecting the lumbar puncture needle with a fine glass pipette in which cerebrospinal fluid was allowed to rise, a method still currently applied [1]. Whereas data from a recent large international, multi-center study on lumbar puncture feasibility that included 3,868 patients confirmed the procedures’ safety [2], complications from lumbar puncture including iatrogenic meningitis, hemorrhage, post-lumbar puncture headache are well recognized [3–6]. In addition to the risks stated above, continuous ICP monitoring includes ventricular catheter-related problems such as cerebral infections, catheter occlusion, or malposition. [7–9]. Financial costs incurred at a single tertiary care institution due to hospitalization for post-lumbar puncture complications were estimated at $20,000 USD/year [10]. To mitigate these risks, various modalities of non-invasive ICP estimation have been described. Of these studies ophthalmodynamometry provided the earliest attempts at non-invasive ICP estimation, they were based on earlier observations by Deyl in 1898 who postulated that papilledema was due to central retinal vein compression where it emerged from the optic nerve into the subarachnoid space in the optic nerve’s dural sheath [11]. To further prove this mechanism, Cushing and Borley experimentally induced papilledema and described the loss of spontaneous venous pulsation due to collapse of the central retinal vein when ICP was raised [12]. Their findings were supported by a canine model, in which the temporal succession of events demonstrated that loss of spontaneous venous pulsation and venous dilation preceded optic disc enlargement was demonstrated [13]. In 1927 Baurmann provided the first evidence of a strong linear correlation between ICP and retinal venous pulse pressure measured by ophthalmodynamometry [14]. His results were replicated by other investigators in a series of animal studies [15–19]. These findings were also reported in human studies by Firsching [20, 21], Motschmann [22] and co-workers. To improve ICP predictive accuracy, Querfurth et al. combined ophthalmodynamometry with simultaneous color doppler of the central retinal and ophthalmic arterial flow velocities. Although the linear correlation between retinal venous pulse pressure and ICP was strong. The combination of ophthalmodynamometry and color doppler parameters was shown to improve the correlation compared to either parameter alone [23]. Intuitively, a strong linear correlation would imply strong predictive accuracy, yet paradoxically these linear models did not achieve a strong predictive power expected from the linear correlation and none of these methods have superseded invasive methods of ICP measurement to date [24–26].

The technique and instrumentation for ophthalmodynamometry was developed by Bajardi around 1906 for the indirect estimation of ocular perfusion [27]. Although this device and its several iterations was superseded by carotid doppler, we have over a series of publications [28–34] described a novel imaging method of Modified Photoplethysmography where a combination of ophthalmodynamometry as a means of generating a range of induced intraocular pressures and slit-lamp imaging of the optic nerve enables modelling of the retinal venous and arterial pulse amplitude and timing characteristics. The physiological basis for the choice of a harmonic regression model in image analysis is to emulate the photoplethysmography wave periodic and non-periodic components [35]. The model consists of a linear spline, which represents the mean of the signal and adjusts for inter-frame image displacement. The first two harmonics of the Fourier series are fitted to the periodic component and a first-order autoregressive error component accounts for the error process. Frequency domain decomposition is performed using custom software, where heat maps of the retinal vascular pulse amplitude distribution are generated and Fourier coefficients of the first two harmonics are extracted. Additional to modelling the retinal arterial and venous systems separately using this method, uniquely, frequency domain analysis allows computationally efficient information filtering and comparative processing of the retinal vascular pulse characteristics [30, 36]. The trade-off in signal resolution between the temporal (Δt) and frequency (Δf) domains is defined by the Heisenberg-Gabor uncertainty principle. It is mathematically expressed as an inequality equation (Δ*f* ⋅ Δ*t* ≥ *C*), where (C) is a constant with a value dependent on how the frequency is measured and conveys the general concept that a non-zero function and its Fourier decomposition cannot be localised to arbitrary precision [37]. Although information between these domains is conserved, unique hemodynamic phenomena can exist solely as a property of the frequency domain such as dispersion (frequency dependent velocity of a wave), impedance (frequency dependent resistance), and the harmonic amplitude distribution. Modified photoplethysmography requires imaging at a slit-lamp, therefore is best applied in an ophthalmology outpatient setting, this excludes the applicability of the method for critically ill patients in the emergency or intensive care setting. A sophisticated handheld system is currently under development by our group, which may make future iterations of the clinical system more versatile for ICP measurement. Although it is possible to perform the test by a single operator, we have used an observer to vocalize the force readings from the ophthalmodynamometer, this allows editing the videos as further detailed in the materials and methods section. Moreover, the technique requires patient cooperation to maintain fixation and remain seated as is required for slit-lamp ophthalmoscopy, this excludes patients with cognitive impairment. A reduced model of the pulsation event is possible from the output of Modified Photoplethysmography it cannot provide information on the hemodynamic pressure-flow wave and although it provides heat maps of the pulse amplitude and timing characteristics, further work is required to analyze vascular geometry specific changes of the pulse characteristics in the Fourier domain. The interaction between ICP, IOP, and the retinal vascular pulse may share characteristics of chaotic systems. In general, systems with at least two of the following properties are considered to be chaotic: bifurcation and period doubling, period three, transitivity and dense orbit, sensitive dependence to initial conditions, and expansivity [38]. Even though Modified Photoplethysmography, unlike other non-invasive methods of ophthalmic ICP estimation, provides continuous vascular observations to potentially resolve this question. The limitation in our work is date is due to the lack of concurrent continuous ICP and IOP measurements during retinal imaging, which particularly the latter, may not be possible out of an experimental / physical model setting. In a recently published study, we described the interaction of the harmonic regression amplitude (HRW_a_) distribution in the retinal vascular system with intraocular and intracranial pressure using a linear mixed effects model. This approach enabled the computation of the variance estimated by these variables in linear space. It was demonstrated that linear interactions of IOP, ICP and the retinal vascular pulse accounted for less than 10% of the variance. This poor explanatory power of a linear model precludes it as a predictor of ICP. This was due to a non-constant variance of the error term of the predictors (heteroscedasticity), which indicated that the correlation was linear within individuals but non-linear between individuals [28]. Therefore, addressing this non-linear component is crucial to achieving high ICP predictive accuracy. In this study, we applied an Extreme Gradient Boost (XGB) supervised machine learning decision tree algorithm in non-invasive ICP estimation from features extracted from the retinal arteries and veins separately. This approach addresses the non-linear component in the data structure and model fit was adjusted through tuning of the hyperparameters. Additionally, parallelization and distributed computing allowed for rapid data computation run times. We hypothesize that the combination of Fourier-domain decomposition of the retinal vascular pulse wave parameters and a regression model derived from a decision tree machine learning algorithm could provide an avenue to non-invasive ICP prediction.

## Materials and methods

### Subject recruitment

Twenty-eight participants were recruited prospectively from the Lions Eye Institute over five years (2015–2020) from referrals made to the clinic for ophthalmic assessment before lumbar puncture for suspicion of idiopathic intracranial hypertension. Study approval was obtained from the University of Western Australia Human Ethics Committee adhering to the tenets of the Declaration of Helsinki. Participants were required to have clear ocular media, no prior history of co-existing retina or optic nerve disease, and were needed to be able to cooperate with the imaging protocol. Written consent was obtained from each of the participants. Lumbar puncture was performed in the lateral decubitus position and measured in centimeter (cm) water. The ophthalmic examination consisted of measurement of visual acuity, Goldmann tonometry, slit-lamp examination, color fundus photography, and modified photoplethysmography. The latter test consists of contact lens ophthalmodynamometry, the purpose was to vary induced intraocular pressure (IOP_i_), with concomitant video imaging of the optic disc.

**Fig 1:**
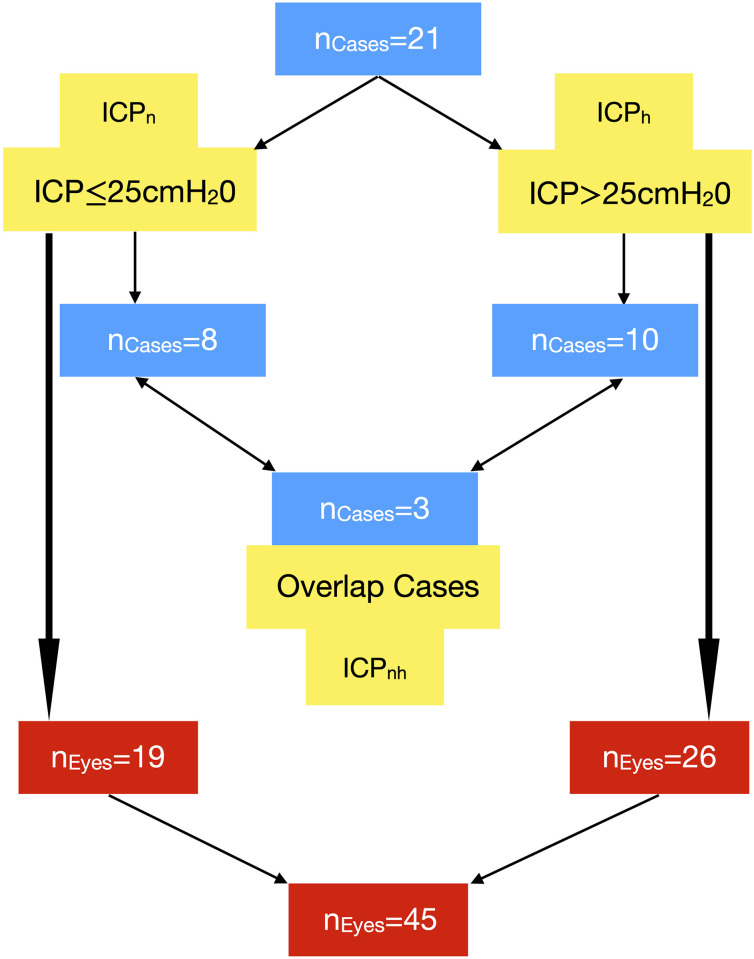
Classification of the study population. Training and test groups study groups, patients with normal intracranial pressure ICP_n_ (ICP≤25cm water) and high intracranial pressure ICP_h_ (ICP>25cm water).

**Fig 2:**
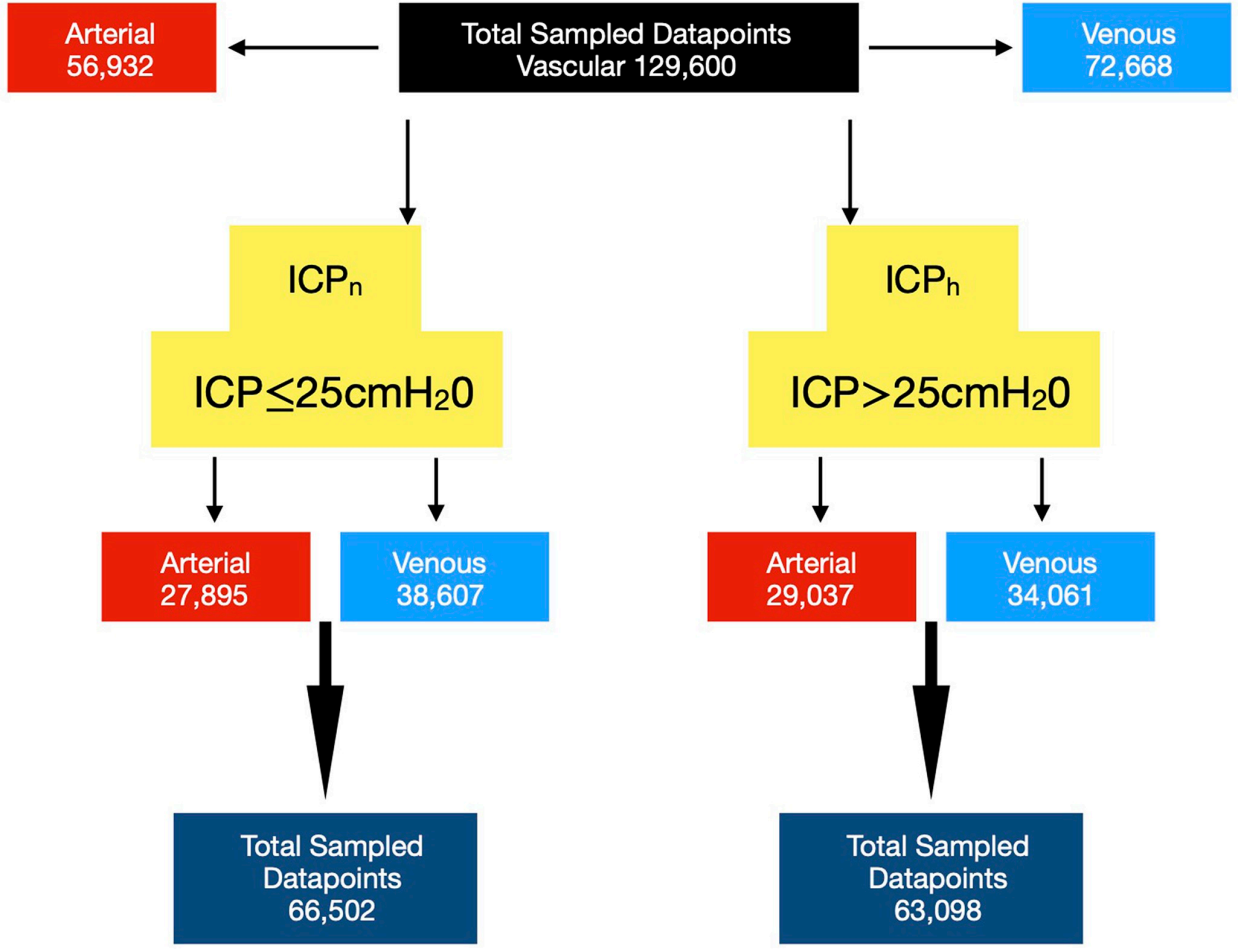
Data points from training and test study groups. The data points are sub-classified into arterial and venous points, in patients with normal intracranial pressure ICP_n_ (ICP≤25cm water) and high intracranial pressure ICP_h_ (ICP>25cm water).

An ICP of 25 cm water was considered the upper normal limit [39], this threshold classified the twenty-one patients in the training and test study groups into ten cases in the high intracranial pressure group (ICP_h_>25cm water) and eight in the normal intracranial pressure group (ICP_n_≤25cm water). Three cases overlapped both groups as a result of interchanging between the ICP_n_ to the ICP_h_ groups over the observation period (Fig 1). A total of 129,600 data points were sampled from the images, 56,932 arterial and 72,668 venous data points (Fig 2). Three eyes were excluded from the analysis due to poor image quality. Additionally, seven subjects were recruited for model validation. data from these cases were not subjected to the training or testing phases of the analysis.

### Image acquisition

Data capture was performed by an operator and an observer in an outpatient setting. While the operator concentrated on the imaging task, the observer vocalized the ophthalmodynamometric force, this was required to create the splice points for video editing. The test requires patient cooperation to sit upright, remain stationary and fixate on a target as required for slit-lamp ophthalmoscopy. Details for the image acquisition workflow is published in earlier work [30]. The optic nerve was imaged under a dynamic range of intraocular pressures using a Meditron ophthalmodynamometer (Meditron GmbH, Poststrasse, Völklingen, Germany). This device consists of a sensor ring, which measures the compression force on the eye. The sensor surrounds a central Goldmann three-mirror fundus contact lens. The ophthalmodynamometric force (ODF) displayed as Meditron units (mu), which were then converted to induced intraocular pressure (IOP_i_) as described by Morgan et al. [40] using the following formula:

$$\begin{array}{c} IOP_{i}=0.89\cdot ODF+IOP_{b} \end{array}$$ (1)

Where IOP_b_ is the baseline intraocular pressure (IOP) in millimeters mercury (mmHg). Video of the optic nerve was captured with an imaging slit-lamp (Carl Zeiss, Germany) with a mounted digital camera (Canon 5D Mark III, Japan). Several sequences of at least three cardiac cycles in length were taken, each at a rate of 25 frames/second. When possible recordings were taken from both eyes. A range of induced intraocular pressure values was between 7–73 mmHg were obtained from each subject. Videos showing motion artifact, reflection from optical media, or decentration of the optic nerve in the image sequence for less than three consecutive cardiac cycles were rejected from the analysis. A pulse oximeter (Nellcor N65, Covidien, Mansfield, MA) was applied to the right index finger; the audio signal from the pulse oximeter was recorded with the video sequence of the optic nerve. This allowed synchronization of the retinal vascular pulse with the cardiac cycle. Timing of the cardiac cycle was generated from the audio signal from the subject’s pulse oximetry recorded on the audio trace of the video segment, which in turn enabled the mathematical analysis of the periodic component from green channel transmittance. A Single high-quality three-cardiac cycle length video recording was extracted from each recording session.

### Image analysis

Image processing was done in Adobe Photoshop CS6, Individual image frames were extracted from each video sequence and saved as Tagged Image File Format (TIFF) files. Each of these images was cropped to an array of pixels. All images from three cardiac cycles were analyzed in R statistical package using custom software [41]. Each data point was represented by the mean of the green channel intensity at time measured as a fraction of the cardiac cycle, rather than in seconds. The periodic trend component was modelled separately for the arteries and veins as a harmonic regression waveform expansion:

$$\begin{array}{c} F\left({f(t)}_{p}\right)=a_{0}+\sum_{n=1}^{\infty }a_{n}\cdot cos\left(n\pi t\right)+b_{n}\cdot sin\left(n\pi t\right)+\epsilon \end{array}$$ (2)

f(t)_p_ = The periodic component of the time series.

a_0_ = Coefficient representing the mean of f(t)_p_.

a_n_ = Coefficient of the cosine function of f(t)_p_.

b_n_ = Coefficient of the sine function of f(t)_p_.

n = Integer 0,1,2… etc representing the harmonic component.

*ϵ* = error term

Higher harmonic frequency model comparisons were conducted using Akaike Information Criterion (AIC). In most eyes AIC preferred models with first and second-order frequencies, therefore the final analysis was limited to the first and second harmonics. A harmonic regression model was fitted to each pixel in the time series and used to quantify the retinal vascular pulse wave parameters including the harmonic regression wave amplitude (HRW_a_). The model includes a Fourier series representation using the first and second harmonics, linear spline non-periodic component, and a first-order autoregressive error component. Timing attributes captured by the custom software include the cardiac cycle time and time to the minimum point of the harmonic regression wave (time to trough) measured in fractions of the cardiac cycle as indicated from the audio pulse oximetry signal. Image analysis and model fit are detailed in previous publications [30, 33, 36].

### Machine learning algorithm-Extreme Gradient Boost (XGB)

Extreme gradient boost algorithm is an ensemble machine learning regression method based on decision trees that use the gradient descent architecture to boost weak learners [42]. Boosting builds decision trees sequentially such that each subsequent tree aims to reduce the errors of the previous tree and the residual errors are then updated. R statistical package [41] was used to generate the model for each vascular system independently. Bayesian optimization was used to tune seven of the model hyperparameters aimed at regulating the model fit. Five-fold cross-validation with ten early stopping rounds was applied in this step. Tuned hyperparameters included lambda (*λ*) L2 regularization term on weights (analogous to Ridge regression), this parameter reduces the influence of outliers by factoring in the denominator of the similarity score (defined as the ratio of the sum of the residuals squared and the number of the residuals). Gamma (*γ*) is the minimum loss reduction required to make a further partition on a leaf node of the tree. The larger gamma is, the more conservative the algorithm. Eta (*η*), the learning rate, is a scalar that determines step size in gradient descent and shrinks the weights on each step. Alpha (*α*) L1 regularization term on weights (analogous to Lasso regression). The maximum depth of the tree (max_depth) is the maximum number of nodes allowed from the root to the farthest leaf of a decision tree, deeper trees can model more complex relationships by adding more nodes. Minimum child weight (min_child_weight) is the minimum number of samples required to create a new node in the tree. Subsample corresponds to the fraction of observations (the rows) to subsample at each step, and nrounds the number of model iterations [42, 43]. Hyperparameters values for each model are listed in Table 1. If parameters are not set, default values are chosen by the XGB algorithm.

**Table 1:** Bayesian optimised Extreme Gradient Boost hyperparameters.

Hyperparameter	Arterial Model	Venous Model
Alpha (*α*)	1	0
Lambda (*λ*)	1	1
Gamma (*γ*)	0	0
Eta (*η*)	1	0.4856858
max_depth	10	10
min_child_weight	21.21737	25
subsample	1	0.5725238
nrounds	35	151

Alpha = L1 regularization term on weights, lambda = L2 regularization term on weights, gamma, controls branch depth via the gain, eta the learning rate, max_depth = maximum depth of the decision tree, min_child_ weight = minimum sum of instance weight (hessian) needed in a child, subsample = the ratio of the training instances, nrounds = the number of model iterations.

**Fig 3:**
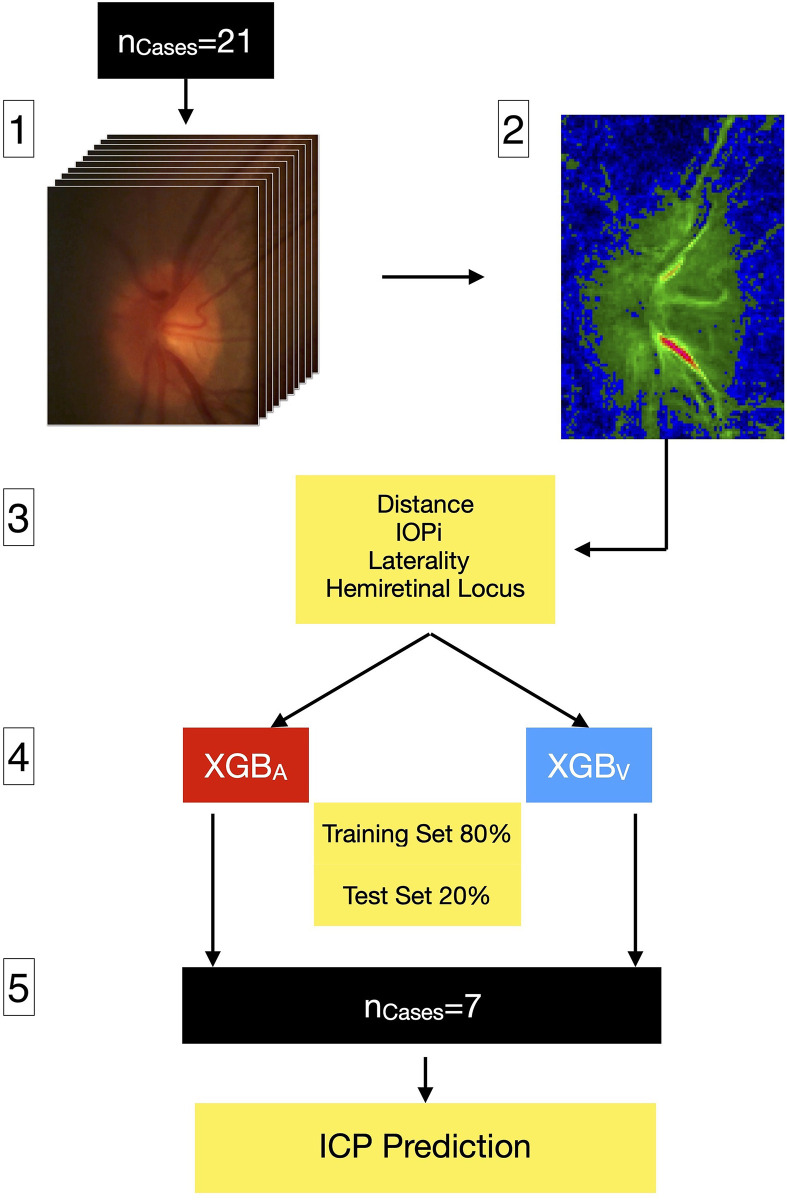
Flow chart of image processing, analysis, and XGB model application. 1) Alignment and segmentation of image frames from a video sequence spanning three consecutive cardiac cycles captured from 21 subjects using Modified Photoplethysmography. 2) Image analysis was performed by fitting a harmonic regression model to each pixel cluster. The periodic component was represented by the first two harmonics of a Fourier series. 3) The Fourier coefficients, harmonic regression amplitude, together with the distance along the vessel, induced intraocular pressure (IOP_i_), ocular laterality (right/left), and hemiretinal locus of the blood vessel (superior/inferior) constituted the model features. 4) Separate retinal arterial (XGB_A_) and venous (XGB_V_) Extreme gradient Boost models were constructed, where an 80/20% split was chosen for the training/test set for each vascular model. 5) Seven hold-out test cases were used for external model validation and intracranial pressure (ICP) prediction.

In our study the training data consisted of nine numerical features (IOP_i_, HRW_a_, the cosine and sine coefficients of the first and second harmonic waves (a_n1,2_), b_n1,2_), hemiretinal location of the vessel (superior or inferior retina), and laterality (right or left eye)). The training labels which were the ICP measured by lumbar puncture in the lateral decubitus position in centimeter (cm) water, were ultimately used to generate the ICP predictions. The training set consisted of 80% of randomly selected vascular pulsation points, the test set consisted of the remaining 20% of the data points. Model parameters were assessed using feature importance, which is a ranking score representing the contribution from the selected feature to the model prediction. It is calculated for a single decision tree by the amount that each attribute split point improves the performance measure, weighted by the number of observations for which the node is responsible. There are three methods for measuring feature importance in XGB, frequency (weight), which is the number of times a feature is used to split the data across all trees. Cover is the number of times a feature is used to split the data across all trees weighted by the number of training data points that go through those splits, and gain is the average training loss gained when using a particular feature at a branching point. Gain, therefore, represents the refinement in accuracy brought by a feature to the branches of the decision tree. XGB divides feature importance by default into two clusters, cluster one contains features of the highest importance to the model, and other features are aggregated in cluster two. To identify the main features driving model prediction, SHAP (SHapley Additive exPlanations) values were calculated, this is an additive feature attribution method that provides a quantitative evaluation of the tree ensemble’s overall impact in the form of particular feature contributions [44, 45]. External validation was achieved using seven hold-out test cases, which were evaluated in a blinded manner. From the hold-out test set mean, median and peak density (defined as the maximum point generated from the distribution of predicted ICPs from each model) of the predicted ICP were compared to the measured ICP. Both Bland-Altman plots and the t-test were used to measure the mean difference between predicted and measured ICP for both the arteries and the veins separately. A flow chart of image processing, analysis, and XGB model application is summarised in Fig 3.

### Statistical analysis

The distribution of the HRW_a_ and the majority of the Fourier coefficients was non-normal, therefore the median was used as a measure of central tendency and the interquartile range (IQR) was used to assess the dispersion of this measure. Where appropriate the mean and standard deviation were reported. The range, minimum, and maximum of these parameters were also computed. When the Levene test was applied to assess the multifactorial homogeneity of variance of the predictors, heteroscedasticity was demonstrated as the assumption of homogeneity of variance was violated (p<0.0001). The Kruskal-Wallis test was used in the hypothesis test of the differences in the medians, and the paired Wilcoxon test with Bonferroni–Holm correction was used for posthoc analysis. Model fit was assessed using R^2^ square, which is a comparison of the residual sum of squares with the total sum of squares. Model Prediction accuracy was estimated by calculating the Mean Squared Error (MSE), defined as the square of the difference between the predicted and actual values of the test set, it assigns more weight to larger errors. Root mean square error (RMSE), which is the standard deviation of the residuals (prediction errors), the higher the number the greater the standard deviation *σ* of the distribution of errors. MSE and RMSE are used to evaluate the influence of outliers on predictions. The mean absolute error (MAE) calculated by the magnitude average difference between the predicted and actual values of the test set was also reported.

## Results

### Descriptive statistics

**Fig 4:**
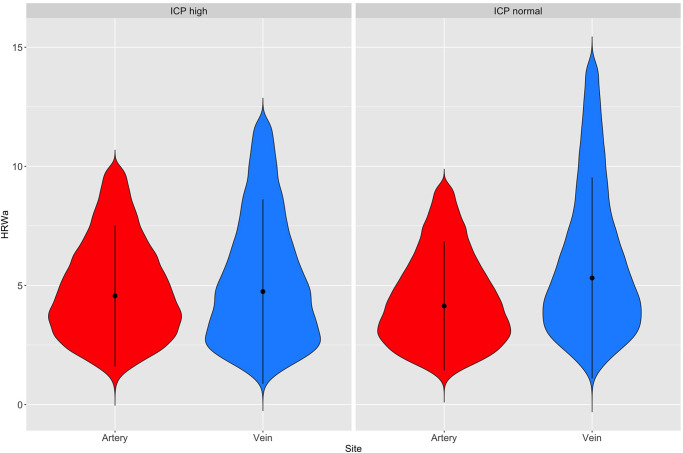
Violin plots of the harmonic regression waveform amplitude (HRW_a_). The central marker in the violin plots indicate the median and interquartile range. Noted are the reduction of the difference in maximum and median retinal vascular pulsation amplitudes within the ICP groups as a consequence of a reduction of the venous and increase in the arterial pulsation amplitudes within the ICP_h_ group. Between group differences indicate a ICP_h_ and ICP_n_ groups.

There were a total of twenty females (95.2%) and one male (4.8%) in the study population. The age demonstrated a bimodal distribution with a mean of 32 years (sd 8.32, range 17–47 years). In the ICP_n_ group median ICP was 18.50 cm water (range 9.50 to 24, IQR = 6), the corresponding values in ICP_h_ group were 31 cm water (range 25.50 to 68, IQR = 10). Table 2 demonstrates the Fourier wave amplitude descriptive parameters in both study groups. Hypothesis tests within ICP group differences were contrasted by vessel type, statistical significance (p<0.0001) was achieved for all Fourier parameters except the b_n2_ coefficient in the ICP_h_ group, which demonstrated no statistically significant difference between the retinal arteries and the retinal veins. Both venous and arterial Fourier wave amplitude descriptive parameters stratified by ICP (between-group differences) demonstrated statistical significance (p<0.001) for all parameters except the retinal venous b_n1_, and arterial a_n2_ coefficients. The ICP_h_ group showed a lower median retinal venous (4.743 vs 5.314) and a higher arterial HRW_a_ (4.559 vs 4.139) compared to the ICP_n_ group (Fig 4).

**Table 2:** Summary descriptive statistics for the high intracranial pressure (ICP_h_) and normal intracranial pressure (ICP_n_) groups.

Parameter	Site	Median	IQR	Min	Max	Range
**ICP_h_**						
**HRW_a_**	Vein	4.743	3.872	0.62	11.983	11.363
**b_n1_**	Vein	-1.418^b^	1.268	-5.6	5.081	10.681
**a_n1_**	Vein	1.079	2.507	-5.519	5.755	11.274
**b_n2_**	Vein	0.114^w^	0.594	-3.723	2.525	6.249
**a_n2_**	Vein	-0.132	0.684	-2.703	2.881	5.585
**HRW_a_**	Artery	4.559	2.958	0.665	9.983	9.317
**b_n1_**	Artery	-1.385	1.047	-4.659	4.096	8.755
**a_n1_**	Artery	0.896	2.381	-4.568	4.485	9.054
**b_n2_**	Artery	0.111^w^	0.655	-2.572	2.606	5.178
**a_n2_**	Artery	-0.110^b^	0.691	-2.169	2.217	4.386
**ICP_n_**						
**HRW_a_**	Vein	5.314	4.218	0.695	14.434	13.739
**b_n1_**	Vein	-1.646^b^	1.511	-6.988	5.148	12.137
**a_n1_**	Vein	1.087	2.675	-5.162	6.727	11.89
**b_n2_**	Vein	0.15	0.598	-3.741	3.417	7.158
**a_n2_**	Vein	-0.173	0.735	-3.07	2.702	5.772
**HRW_a_**	Artery	4.139	2.712	0.75	9.233	8.483
**b_n1_**	Artery	-1.276	1.048	-4.432	3.843	8.275
**a_n1_**	Artery	0.64	2.148	-3.924	4.103	8.027
**b_n2_**	Artery	0.093	0.545	-2.291	2.248	4.539
**a_n2_**	Artery	-0.086^b^	0.663	-2.172	2.133	4.305

HRW_a_=harmonic regression wave amplitude. a_n1,2_=first and second Fourier cosine coefficient, b_n1,2_=first and second Fourier sine coefficients. Min = minimum, Max = maximum. Superscripts denote that hypothesis tests did not achieve statistical significance for within(x^w^) groups ie, between the vascular systems of a single ICP group, and between (x^b^) groups ie, between the same vascular systems of both ICP groups. Descriptive statistics are summarised graphically in Fig 4.

### Machine learning model

#### Model fit and feature importance

**Fig 5:**
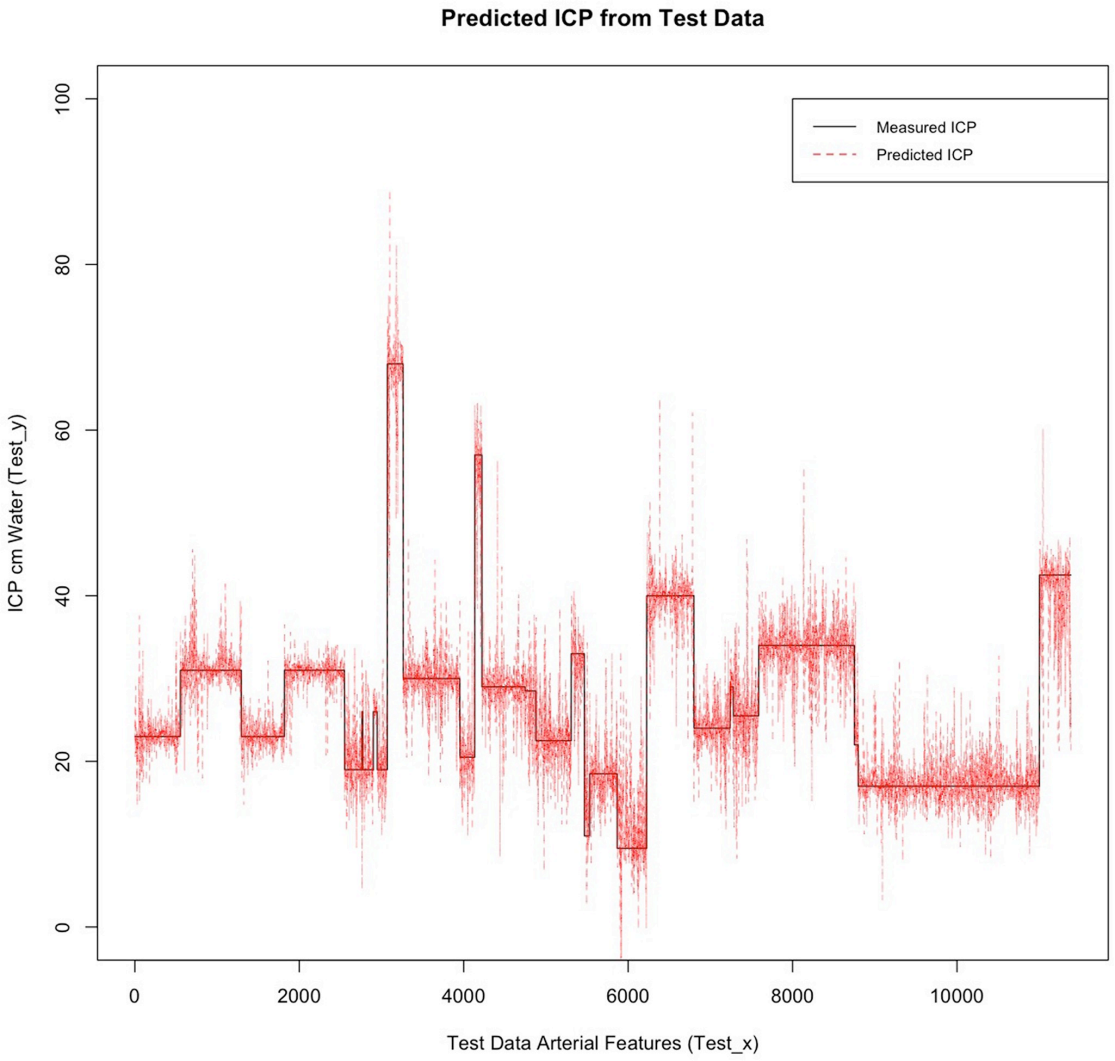
Retinal arterial model fit. The arterial model consisted of a total of 137 nodes, 136 edges, and 7,951 leaves. The model had an R^2^ of 0.89, and other accuracy parameters were: MSE = 10.99, MAE = 2.03, RSME = 3.32.

**Fig 6:**
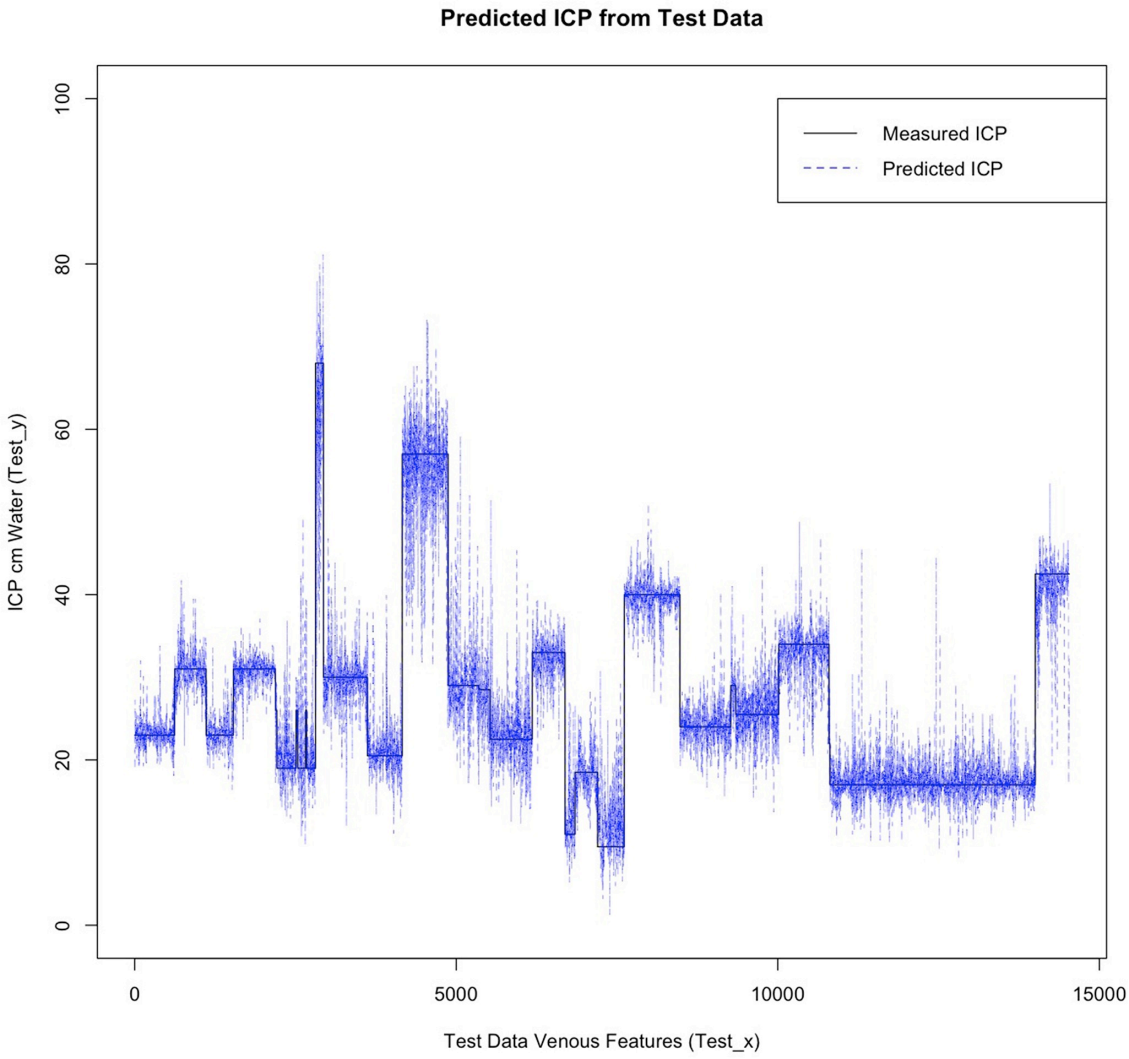
Retinal venous model fit. The venous model was composed of a total of 451 nodes, 450 edges, and 35,589 leaves. The model had a higher R^2^ of 0.91, other accuracy parameters were: MSE = 11.85, MAE = 2.11, RSME = 3.44.

The model fit was was comparable for the arterial and venous data as indicated by an R^2^=0.89 and R^2^=0.91 respectively. Other accuracy parameters were similar for the arterial (MSE = 10.99, MAE = 2.03, RSME = 3.32) and the venous model (MSE = 11.85, MAE = 2.11, RSME = 3.44). The venous decision tree was more complex than the arterial, whereas the venous model was composed of a total of 451 nodes, 450 edges, and 35,589 leaves, the arterial model consisted of a total of 137 nodes, 136 edges, and 7,951 leaves. Model complexity and fit of the arterial and venous models are demonstrated in (Figs 5 and 6). The weighted cover in the figures represents the distribution of the average weighted number of residuals clustered in leaves at a certain depth of the decision tree.

**Fig 7:**
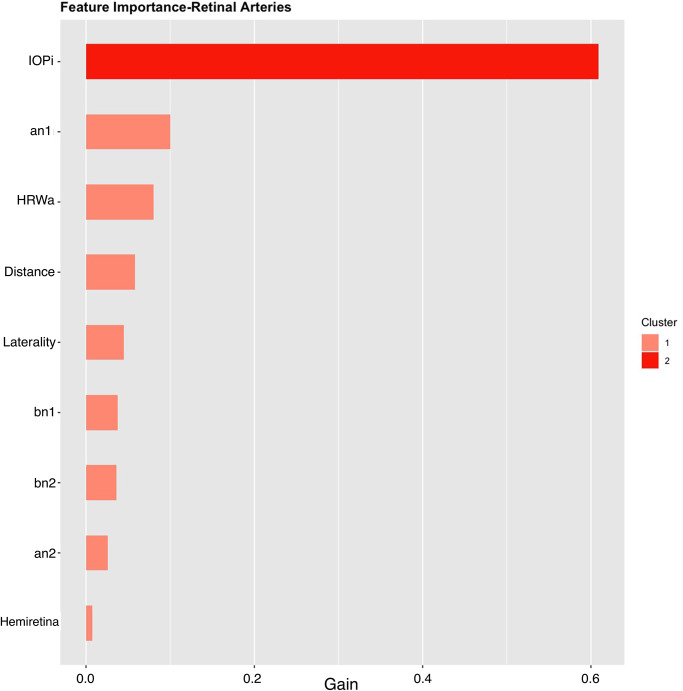
Importance plot retinal arterial model. IOP_i_, a_n1_ and HRW_a_ were the most important features in this model. IOP_i_ = Induced intraocular pressure, HRW_a_=Harmonic regression wave amplitude, a_n1,2_ = the cosine coefficient of the first and second harmonics, b_n1,2_ = the sine coefficient of the first and second harmonics, laterality = left / right eye, Distance = distance along the retinal vessel measured in mm, hemiretina = superior / inferior retina.

**Fig 8:**
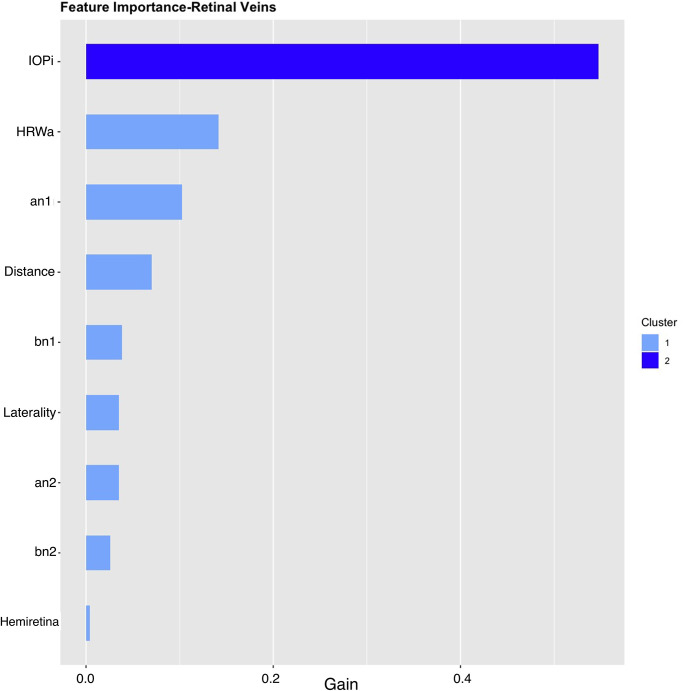
Importance plot retinal venous model. IOP_i_, HRW_a_, and a_n1_ were the most important features in this model. IOP_i_ = Induced intraocular pressure, HRW_a_=Harmonic regression wave amplitude, a_n1,2_ = the cosine coefficient of the first and second harmonics, b_n1,2_ = the sine coefficient of the first and second harmonics, laterality = left / right eye, Distance = distance along the retinal vessel measured in mm, hemiretina = superior / inferior retina.

Global feature importance ranks the nine features of each vascular model by the feature gain, cover and frequency used in the prediction of ICP (Figs 7 and 8). For the arterial model these were IOP_i_ (0.6092), a_n1_ (0.1000) and HRW_a_ (0.0804), similarly for the venous model IOP_i_ (0.5476) and a_n1_ (0.1024), dominated the feature importance, however unlike the arterial model HRW_a_ (0.124) showed higher importance compared to a_n1_ coefficient. When feature frequencies of the arterial and venous models were compared IOP_i_ (0.2547 vs 0.2028), a_n1_ (0.1357 vs 0.1362) and HRW_a_ (0.1228 vs 0.1291), accounted for approximately 51–47% of each model’s feature importance respectively (Table 3).

**Table 3:** Feature importance of the machine learning models.

Feature	Gain	Cover	Frequency
**Arterial Model**			
IOP_i_	0.6092	0.3207	0.2547
a_n1_	0.1000	0.0782	0.1357
HRW_a_	0.0804	0.2165	0.1228
Distance	0.0585	0.0821	0.1309
Laterality	0.0450	0.0112	0.0310
b_n1_	0.0378	0.1110	0.1037
b_n2_	0.0360	0.0917	0.1101
a_n2_	0.0258	0.0828	0.0961
Hemiretina	0.0073	0.0057	0.0150
**Venous Model**			
IOP_i_	0.5476	0.2673	0.2028
HRW_a_	0.1414	0.2094	0.1291
a_n1_	0.1024	0.1123	0.1362
Distance	0.0701	0.1185	0.1384
b_n1_	0.0383	0.0810	0.1115
Laterality	0.0352	0.0082	0.0254
a_n2_	0.0349	0.1083	0.1218
b_n2_	0.0261	0.0890	0.1175
Hemiretina	0.0041	0.0060	0.0173

Gain is the difference between the calculated similarity scores for successive leafs in the decision tree, it represents the average training loss gained when using a feature for further branching. Cover the number of times a feature is used to split the data across all trees weighted by the number of training data points that go through those splits Frequency represents the ratio of the number of times a feature is used to split the data across the whole tree. IOP_i_, a_n1_ and HRW_a_ dominated the feature importance of both models. Feature importance is demonstrated graphically in Figs 7 and 8.

#### Model SHAP values

**Fig 9:**
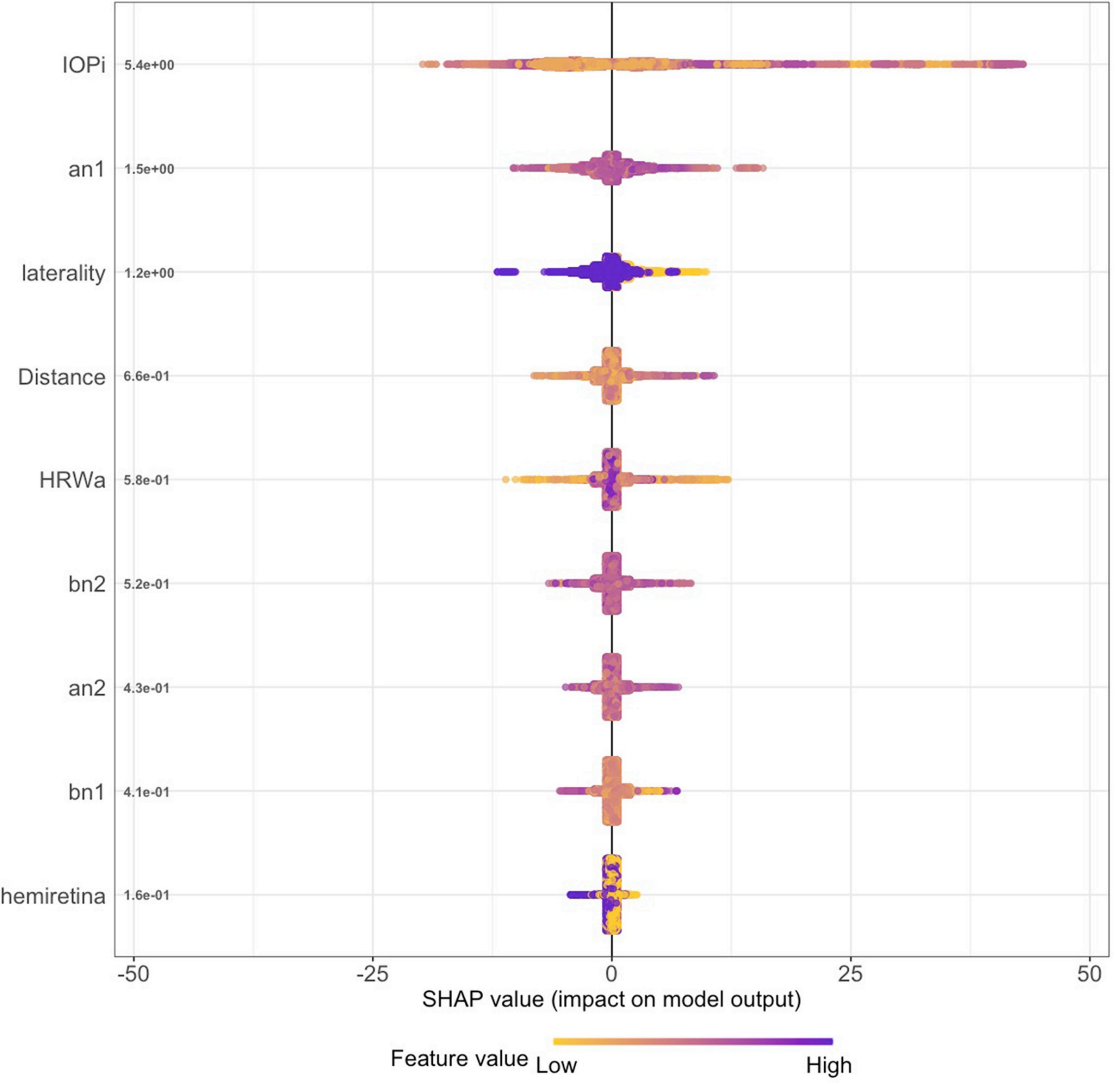
SHAP summary plot retinal arterial model demonstrating the feature contribution of the XGB model predicting ICP from the arterial model. Induced intraocular pressure (IOP_i_) was the most important feature in the model (mean SHAP = 5.3884), approximately four times the value of the cosine coefficient of the first harmonic (a_n1_ mean = 1.4689). Laterality = Right/Left eye, Distance = Retinal vascular pulsation amplitude as a function of distance from the center of the optic disc in mm, HRW_a_=Harmonic regression wave amplitude, a_n1,2_, b_n1,2_=cosine and sine coefficients of the first and second harmonics, hemiretina = Superior/Inferior retina.

**Fig 10:**
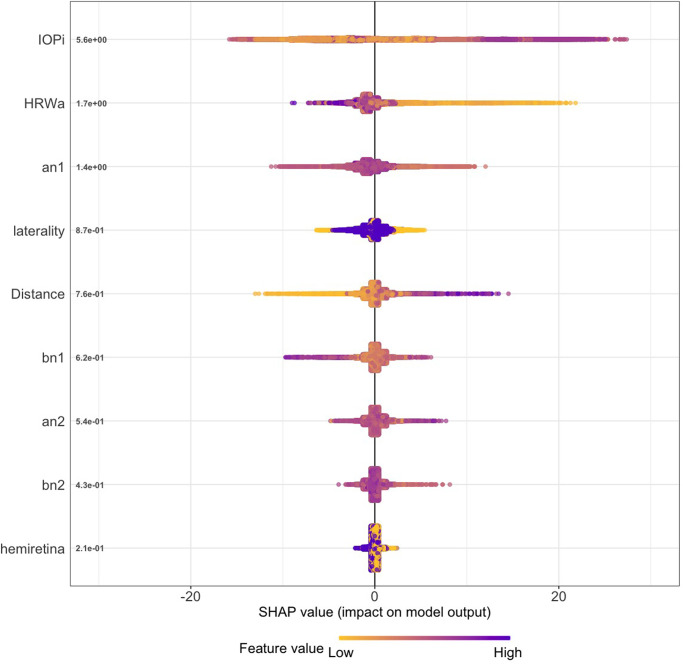
SHAP summary plot retinal venous model demonstrating the feature contribution of the XGB model predicting ICP from the venous model. Induced intraocular pressure (IOP_i_) was the most important feature in the model (mean SHAP = 5.723), approximately four times the value of the harmonic regression wave amplitude (HRW_a_ mean = 1.425). HRW_a_=Harmonic regression wave amplitude, a_n1,2_, b_n1,2_=cosine and sine coefficients of the first and second harmonics, laterality = Right/Left eye, Distance = Retinal vascular pulsation amplitude as a function of distance from the center of the optic disc in mm, hemiretina = Superior/Inferior retina.

The SHAP summary plot (Figs 9 and 10) combines feature importance with feature effects, therefore it allows to explore interactions between features for the predicted variable. It is important to consider that SHAP values do not identify causality [45, 46]. Four properties can be derived from the SHAP summary plot:

1. Feature importance: features are ranked on the y-axis in descending order according to their importance in the prediction of ICP from each vascular model.
2. Impact: SHAP measures the impact of variables taking into account the interaction with other variables of the model. The horizontal location shows whether the effect of that value is associated with a higher or lower prediction. This is accomplished by calculating the importance of a feature by comparing what a model predicts with and without the feature in every possible combination. The x-axis measures the SHAP value, which indicates the change in model output in log-odds.
3. Value: The color scale indicates whether that variable is high (blue) or low (yellow) for that observation where every point represents a row from the original dataset. Overlapping points are jittered in the x-axis direction. It should be noted that categorical variables were numerically encoded for the analysis (left/inferior = 0, right/superior = 1) for the laterality and hemiretinal location respectively giving a binary nature to the color scale.
4. Correlation: Indicated by the relation between impact and color scale, a positive correlation occurs when high values of the feature have a positive predictive impact.

From both plots, it can be observed that the impact of IOP_i_ on the model prediction was dependent on the value and showed a positive correlation, low IOP_i_ values had a low predictive impact, and higher IOP_i_ values had a positive impact on prediction, particularly for the venous model. This feature also showed higher dispersion than any of the tested features. The hemiretinal location of the tested vessel showed the lowest impact on model predictability. Laterality and distance of the data point along the vessel measured from the center of the optic disc attained more significance compared to the feature importance without variable interactions (Table 3), particularly for the arterial model, where there was a correlation with laterality, right eyes had negative and left eyes a positive impact on prediction, the venous model did not demonstrate this effect. Pulsation values obtained from vascular points in proximity to the optic disc had a less predictive impact and pulsation values from more peripheral locations in the vessel had a higher impact on model prediction (positive correlation).

The arterial model showed no correlation with HRW_a_ values, furthermore, this feature was less significant when interactions were considered. In contrast, the venous model demonstrated a negative correlation, and it retained its significance both with and without variable interactions (Table 3).

The a_n1_ Fourier coefficient had the highest feature importance of all coefficients in both models, and other coefficients had a low rank. The correlation of the coefficients was different for each model. Whereas the arterial model demonstrated that the a_n1,2_, and b_n1,2_ showed low positive correlation on model predictability, the venous model on the other hand showed that a_n1,2_ and b_n1,2_ had opposite correlation on predictability, where a_n1_, b_n1,2_ values had a negative correlation and a_n2_ had a positive correlation.

Mean SHAP values are listed in Table 4, where IOP_i_ demonstrates the highest mean SHAP values in both vascular models (arterial = 5.3884 and venous = 5.6375), this was approximately four times the mean value of a_n1_ (arterial = 1.4689 and venous = 1.3856) and the others among the three most significant features (arterial laterality = 1.2477, venous HRW_a_=1.7024).

**Table 4:** Mean SHAP values for the arterial and venous models.

Feature	Mean SHAP Value
**Arterial Model**	
IOP_i_	5.3884
a_n1_	1.4689
Laterality	1.2477
Distance	0.6626
HRW_a_	0.5754
b_n2_	0.5171
a_n2_	0.4275
b_n1_	0.4082
Hemiretina	0.1563
**Venous Model**	
IOP_i_	5.6375
HRW_a_	1.7024
a_n1_	1.3856
Laterality	0.8710
Distance	0.7625
b_n1_	0.6178
a_n2_	0.5370
b_n2_	0.4284
Hemiretina	0.2103

SHAP values are based on a game theoretic approach to estimate the contribution of a feature to the models prediction by considering all possible combinations of the feature to the outcome in what is called a power set. SHAP values are demonstrated graphically in Figs 9 and 10.

### Model external validation

**Fig 11:**
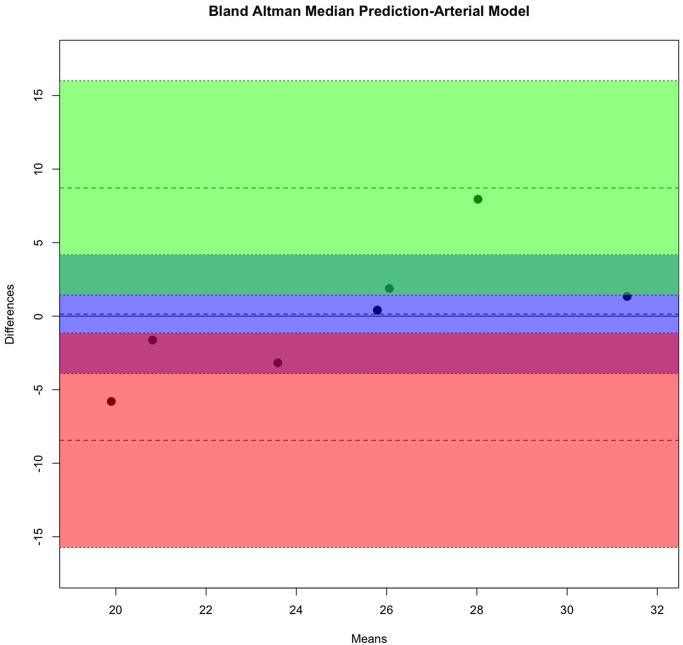
Bland-Altman plot predicted median predicted intracranial pressure of the arterial Extreme Gradient Boost model. The intervals of two standard deviations are considered as the concordance limits between the two measurements, accounting for 95% of the observed differences. The Bland-Altman bias was 0.139±1.6545 cm water (p<0.94), the arterial model provided a potential avenue for internal validation of the prediction.

**Fig 12:**
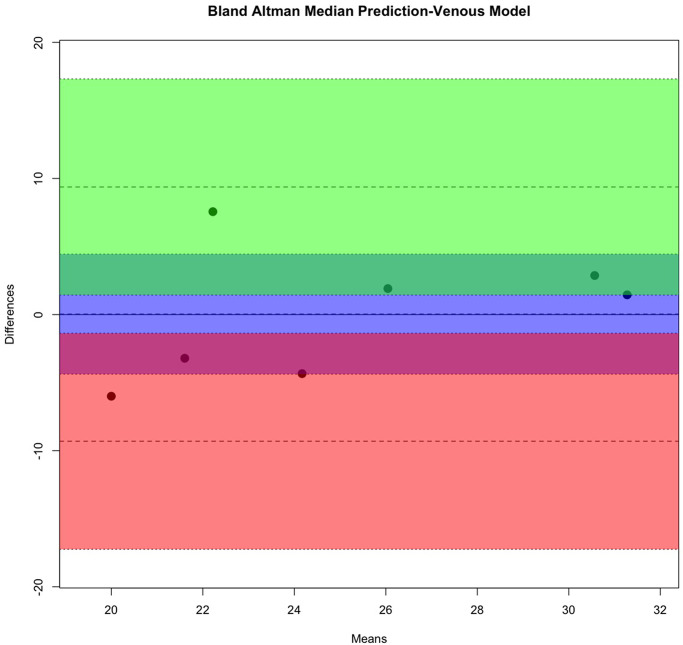
Bland-Altman plot predicted median predicted intracranial pressure of the venous Extreme Gradient Boost model. The intervals of two standard deviations are considered as the concordance limits between the two measurements, accounting for 95% of the observed differences. The Bland-Altman bias from the venous model (0.034±1.8013 cm water (p<0.99)) was lower compared to that of the arterial model.

A group of seven cases was used as the holdout test set, in this group all cases were females. Four cases (57.1%) had an ICP > 25cm water. The median ICP in this group was 29.5cm water (range 26 to 32, IQR = 5.25). The remaining three cases (42.9%) had a median ICP of 20cm water (range 17 to 22, IQR = 2.5). Table 5 summarises the mean, median, and peak density of the predicted ICP from each vascular model. When the mean, median, and peak density for predicted ICP from the arterial and venous models are compared using the t-test and Bland-Altman bias statistic (Table 6), it is clear that the predicted ICP estimated from the venous median had the best agreement with measured ICP as indicated by the lowest Bland-Altman bias. A comparison of measured and median estimated ICP is demonstrated graphically in Bland-Altman plots (Figs 11 and 12).

**Table 5:** Hold-out test set comparing measured and predicted ICP (cm water) using the XGB models for both the arteries and veins.

Case	ICP	Mean (se)	Median (IQR)	Peak Density
		**Arterial Model**		
1	22	26.59 (0.17)	25.18 (8.54)	24.98
2	32	24.13 (0.32)	24.05 (6.47)	24.45
3	20	24.52 (0.30)	21.63 (8.94)	20.07
4	32	31.86 (0.26)	30.66 (10.79)	28.70
5	17	23.26 (0.16)	22.80 (7.06)	21.36
6	27	28.25 (0.23)	25.12 (8.49)	24.05
7	26	28.65 (0.41)	25.59 (13.79)	23.38
		**Venous Model**		
1	22	27.77 (0.19)	26.34 (8.26)	24.47
2	32	30.90 (0.34)	29.13 (9.09)	27.77
3	20	24.18 (0.21)	23.21 (6.24)	22.98
4	32	31.29 (0.25)	30.55 (9.73)	30.20
5	17	22.78 (0.13)	23.00 (6.22)	23.33
6	27	26.26 (0.11)	25.09 (6.97)	24.01
7	26	20.70 (0.25)	18.44 (8.21)	15.91

Three Methods were used to assess the best estimate the mean, median and peak density of predicted ICP.

**Table 6:** Bland-Altman analysis and t-test, comparing between the measured and predicted ICP using XGB.

Predicted ICP	t-test statistic	p-value	Bland-Altman Bias (sd/se)
**Arterial Model**			
Mean	-0.9036	0.4011	-1.6086 (4.7101/1.7803)
Median	0.0838	0.936	0.1386 (4.3775/1.6545)
Peak density	0.8352	0.4356	1.2871 (4.0775/1.5412)
**Venous Model**			
Mean	-0.7098	0.5045	-1.1257 (4.1963/1.5861)
Median	0.0190	0.9854	0.0343 (4.7657/1.8013)
Peak density	0.5069	0.6303	1.0471 (5.4659/2.0659)

The measured ICP was compared against the mean and peak density of the estimate from both vascular models. The venous median demonstrates the highest agreement with measured intracranial pressure. sd = standard deviation, se = standard error. Bland-Altman plots are displayed in (Figs 11 and 12).

## Discussion

Extreme Gradient Boost demonstrated favorable accuracy in the non-invasive prediction of ICP applied to Modified Photoplethysmography data. Quantitative interference due to optical reflections, shadowing, blink, and motion artifact secondary to saccadic movements render ophthalmic imaging artifact prone. Modified Photoplethysmography controls these multiple sources of interference. The Goldmann contact lens used for optic nerve observation and imaging eliminates blinking and reduces motion artifact providing optical continuity, field stability, and reducing information degradation. Induced intraocular pressure generates a range vascular pulse amplitude responses, therefore, enabling comparative analysis of the pulse wave under a range of transmural pressures. Moreover, retinal vascular pulse wave decomposition in the Fourier domain allows for computationally efficient information filtering. The harmonic regression approach applied in image analysis not only adjusts for motion artifacts through its linear spline; it applies a statistical approach to evaluate the fit of the Fourier harmonics to the non-periodic component of the vascular pulse. Hence it facilitates the decision of rejecting an analysis output where the signal model fit does not achieve statistical significance at each image pixel cluster [30, 36].

Current clinical ophthalmic literature cites other approaches to non-invasive ICP prediction, these can be classified into clinical/retinal imaging (pupillometry, IOP, optical coherence tomography (OCT), fundus photography, ophthalmodynamometry), radiological (Ultrasonography (U/S)/Computed Tomography (CT)/Magnetic Resonance Imaging (MRI)), and electrophysiological methods (Flash Visual Evoked Potentials (VEP)). The accessibility of the pupil and the development of objective quantitative hand-held modalities to measure pupillary diameter and function have made it an appealing option for the non-invasive estimation of ICP in an intensive care unit (ICU) setting. In the largest multi-center study to date, Chen et al. used the NeurOptics pupillometer to correlate the pupillary function with ICP in a population of 134 ICU patients. They described the temporal inverse relationship of pupillary reactivity with raised ICP, which they termed the Neurological Pupil index, which is derived by algorithmically transforming parameters involved in the pupillary light reflex. Interestingly, this approach had forecasting capability as the authors reported that an abnormal Neurological Pupil index preceded an ICP spike on average by 15.9 hours [47]. However, this approach yielded a course range of ICP values correlated with pupillary function. In a prospective observational study, Stevens et al. found a weak but statistically insignificant relationship between the Neurological Pupil index and ICP [48]. Hence, further research is required to establish the role of automated pupillometry in ICP estimation.

Tonometry has demonstrated inconsistent results in ICP prediction. Sajjadi et al. measured IOP using a Schiotz tonometer in 50 subjects who underwent lumbar puncture. They reported a strong correlation (R = 0.955, p<0.001) independent of body mass index, age, and neurological diagnosis [49]. Other investigators failed to replicate these results using different techniques of IOP estimation [50–54].

Over the last two decades, OCT has been central to the diagnosis and management of optic nerve disorders, the earliest report was by Borchert et al. who patented a method to estimate ICP using OCT measurements of RNFL thickness; however, the authors do not provide the correlations between these variables necessary generate a prediction [55]. A multitude of parameters have been evaluated for potential estimation of ICP [56–60]. In a multicenter study, Vijay et al. reported that optic nerve head central thickness was found to be the most closely associated parameter with ICP (R = 0.60–0.73) among a variety of macular and optic nerve protocols [61]. However, the association between the many OCT parameters, papilledema severity, and ICP is complex and remains undefined. Moreover, due to the need for subject cooperation, this test cannot be applied to patients with severe neurological disorders. To address this limitation, Andersen et al. used the retinal arterio-venous ratio as a biomarker of elevated ICP. Images were recorded using an Epicam portable camera, this method achieved a 94% (85–98%) sensitivity and 50% (34–66%) specificity in detecting patients with an ICP ≥ 20mmHg. Indicating that although there was a 94% probability of correctly identifying individuals with ICP ≥ 20 mmHg, this was mitigated by the 50% probability of misclassifying healthy individuals [62], thereby limiting the practical applicability of this approach.

Radiological imaging of retinal vascular parameters has been explored as a substitute for clinical methods especially since practicality demands the ability to predict ICP with minimal patient cooperation in the ICU setting. In a prospective case-control study, Jeub et al. used transbulbar sonography for the measurement of vascular flow in the central retinal artery. At a threshold value of 11.0 cm/s, the peak systolic velocity predicted pathological ICP levels with a 70% sensitivity and 69% specificity [63]. Using spectral Doppler imaging, Miller et al. reported the reduction in blood flow velocity in both the central retinal artery and central retinal vein in 18 children with elevated ICP (p<0.02), however, the limited number of recruited subjects for this study precluded the calculation of statistical test accuracy parameters [64]. Ragauskas et al compared a two-depth transorbital Doppler technique to measure blood flow velocity in the ophthalmic artery with ultrasonographic measurement of optic nerve sheath diameter [65], this method was reported to have better diagnostic reliability for detecting elevated ICP compared to optic nerve sheath diameter measurement [66–69]. An independent clinical validation study determined that this technique had a fair agreement to ICP measured using lumbar puncture [70]. The theoretical basis establishing a correlation between optic nerve sheath diameter and ICP was first suggested in 1968 by Hayreh et al. in a primate model [71]. Subsequent studies have imaged this parameter using U/S, CT, and MRI making it a favorable option for subjects with significant neurological impairment. In the largest study to date, Rajajee et al. found the optimal optic nerve sheath diameter for detection of ICP > 20 mmHg was >0.48 cm as measured by U/S, where a sensitivity of 96% and specificity of 94% were achieved [72]. Most studies indicate an optic nerve sheath diameter of >5 mm as the threshold for determining elevated ICP [73–75]. Measurement of the optic nerve sheath diameter using CT or MRI can overcome this limitation. High agreement and reproducibility have been reported between these imaging modalities [76–78]. However, radiological methods have high operational costs, and image interpretability is operator dependent particularly in the case of U/S. Some disorders such as subarachnoid hemorrhage may not be suited for this modality [79]. Moreover, the range of pressure correlation with optic nerve sheath diameter can be narrow, the iCOP study reported a favorable correlation between optic nerve sheath diameter and ICP between 3.7 mm Hg and 26.5 mm Hg [80]. The precision and accuracy of MRI measurements of optic nerve sheath diameter are yet to be defined, as well as the optimal measurement technique and the influence of the time course of ICP fluctuations on changes in optic nerve sheath dimension [81].

Flash Visual Evoked Potentials (FVEPs) can demonstrate the integrity of the visual pathway from the retina to the occipital cortex. A longer FVEP wave crest latency, a reduction in amplitude, and an increase in wave width have been observed with elevated ICP. These findings were reported from two early studies where a relatively strong linear relationship (R^2^ ≈ 0.7) between FVEP N2 wave latency and ICP was observed. Using this method high correlation was particularly demonstrated at ICP levels >300 mm water [82, 83]. These findings were replicated using either a single or combined modality with transcranial doppler [84, 85]. There are significant limitations with FEVPs, a significant level of expertise is required to administer the test, it is unsuitable in patients with bifrontal lobe pathology, retinal damage, or optic neuropathy [84]. Moreover, factors such as blood glucose concentration, the patient’s nerve conduction rate, and electrolytes levels can result in high variance in the FVEP waveform properties [86].

Early studies which applied ophthalmodynamometry to this research question correlated retinal venous pulse pressure as a single parameter with ICP [20–22]. The wide range of linear correlations (R = 0.69–0.968) did not, however, yield a clear conclusion regarding the applicability of their findings. Further attempts to improve predictability such as seeking an optimal reliability cutoff point or excluding patients with papilledema either did not produce the intended practical outcome or further restricted its applicability [21]. Querfurth et al. introduced simultaneous color doppler monitoring of the central retinal and ophthalmic arterial flow velocities to improve predictability. They reported a more significant correlation (R = 0.95, p < 0.005) with combined parameter approach [23]. Modified photoplethysmography provides a quantitative ICP prediction along a continuous scale. Fourier domain decomposition of the retinal vascular pulse amplitude enables a selective inclusion of pulse wave harmonics hence, increasing the signal-to-noise ratio in the data set. In a recent study, we described a physiological model to estimate ICP where a plot of the induced intraocular pressure (x-axis) against the retinal venous pulse (y-axis) was measured using Modified Photoplethysmography. Predicted ICP was plotted from the x-axis intersection extrapolated from the peak retinal venous pulsation amplitude. A mean absolute error of 3.0 mmHg was achieved using this technique [29]. However, the XGB approach adds unique advantages: 1) The machine learning decision tree model addresses the heteroscedastic data structure directly and allows the model fit to be optimized as cases are added to the dataset thereby futureproofing the model’s performance. 2) An improved predictive analysis by a reduction in the mean bias to 0.034±1.80 cm water (0.025±1.32 mmHg). 3) The ability to generate an independent prediction from the retinal arteries and veins and from all points from the retinal vessels in the image field enables visualization of the distribution of the predictions in the form of a peak density plot and to draw comparisons from the independent outputs. Therefore the XGB approach provides a method of internally validating the prediction from each case. However, papilledema may reduce the predictive accuracy when the renormalization of the ICP is followed by a lag in regression of the optic nerve changes [87], this may impact diagnostic accuracy of post-treatment serial Modified Photoplethysmography imaging tests.

Artificial intelligence classification methods that use clinical, electrophysiologic, or radiographic data to discriminate between normal and high ICP have been described [88–90]. Neural network classification models have yielded a total accuracy ranging between 70.2±4.5% to 92.05±2.25%. Golzan et al. estimated ICP using a neural network regression model derived from retinal venous pulse amplitude measured using the Dynamic Vessel Analyser, which is the only commercially available device used to quantitatively measure the retinal vascular pulse parameters. This device uses arbitrary units rather than frequency domain decomposition of the pulse wave. They reported a mean square error of ±1.27 mmHg in ICP prediction. However, they did not validate the model predictions on external cases, therefore, only internal validation results were reported. Moreover, only the venous pulse amplitude was used in the model prediction the arterial pulse characteristics was not taken into consideration in the model. In contrast to our method, the pulse amplitude was quantified using empirical units rather than a frequency domain decomposition strategy [91]. In our study, the XGB approach provided unique advantages over other machine learning options, hyperparameter tuning using Bayesian optimization (Table 1), mitigated model overfitting, this is the case where model output is not generalizable due to close fit to the sample data, a known limitation in decision tree machine learning algorithms. The wide normal ICP range (2–25 cm water) [39, 92] and the predominance of non-linear dynamics in the interactions between ICP, IOP, and the retinal vascular pulse [28] may both contribute to data heteroscedasticity, which is the non-constant distribution of the error term of the predictors. This was addressed by the unique ability of a decision tree regression to generate a prediction without specifying an average structure for the model. The specific pruning algorithm is a significant factor in addressing the heteroscedasticity [93]. Moreover, the XGB analysis approach requires minimal data pre-processing and neither normalization nor scaling are necessary [94, 95].

Intracranial pressure predictions derived from the venous model demonstrated better agreement with measured ICP compared to that provided by the arterial model. This may be due to the higher amplitude pulsation in the retinal veins compared to the arteries and the difference in venous pulsation amplitude between the ICP_n_ and ICP_h_ groups. Functional and structural differences between the arterial and venous systems, which in turn result in differences in wall tension and compliance may have played a role. Blood vessel walls consist mainly of water (70%), which is inelastic and incompressible, the remaining structure consists of a mesh of fibers with elastic properties. The fibrillary material consists of collagen, elastin, and smooth muscle cell layer in various percentages depending on the type and location of the vessel [96, 97]. Heterogeneity of structural building blocks of the vessel wall results in a radius-tension curve with non-linear characteristics. Whereas collagen fibers demonstrate a steep length-tension relationship and are closer to the linear relationship as expected from Hooke’s law, elastin has a flat length-tension relationship [98], and that of smooth muscle is modified by the level of contraction [96]. Additionally, there are geometric factors that augment the venous response to changes in transmural pressure, at physiological ranges, a relatively small increase of transmural pressure from 0 to 10 mm Hg increases the venous volume by ∼ 200%, which reflects a change in geometry from ellipsoidal to circular associated with an increased cross-sectional area. At supra-physiological venous transmural pressure (>40mmHg) there is a minimal increase in the relative volume as a result of the increase in compliance. In contrast, the arterial wall shows a smaller slope of the gradient between relative volume and transmural pressure accounting for the higher tolerance to transmural pressure [98]. Therefore, the arterial wall has curvilinear compliance over a range of transmural pressures, whereas venous walls have a semi-sigmoidal range of compliance. These structural and consequently functional differences may underlie the differences in vascular response to raised ICP and consequently may impact model predictive accuracy. Models derived from each vascular system misclassified one of the hold-out test cases with high ICP (case-2 in the arterial model and case-7 for the venous model). Additionally, both models predicted raised ICP for case 1 when the measured ICP was normal. The agreement between the predictions (≥25 cm water) derived from the XGB models from both vascular systems in case-1 (Table 5) raises the possibility that the predicted ICP may represent the actual ICP. This scenario highlights the challenges of lumbar puncture manometry. Studies based on continuous monitoring report that ICP generally measures between 5 -10 mm Hg above atmospheric pressure [99]. However, this value may fluctuate within a range of 30 cm water over 12 hours [100]. Therefore, a time delay between the lumbar puncture and the ophthalmic assessment may have resulted in a spurious outcome in this case. Furthermore, a precise ICP estimate requires a consistent manometry technique where the needle entry point needs to be on the same level as the midline of the spine, which should also be at the same level as the patient’s head. To complicate matters leakage around the needle can result in an erroneous estimate [6]. Further consideration needs to be given to the fact that ICP is estimated and measured from different levels. A historic assumption intracranially measured ICP (EVD-ICP) is equal to opening pressure measured at the lumbar spine (LP-ICP) [101, 102]. In recent years, this matter has been a subject of debate where some studies have documented agreement between intracranial and lumbar measurements [103], correlated [104, 105], and others have disputed both agreement and correlation [106, 107]. Among these studies, Lenfeldt et al. experimentally raised the ICP using computerized lumbar infusion in subjects with communicating hydrocephalus. Simultaneously measured LP-ICP and Brain tissue ICP demonstrated a strong correlation (R^2^ = 0.98) between the two ICP measurements throughout a pressure range of 0–600 mm water, the mean±standard deviation was -10±29 mm water. Therefore, LP-ICP correlates strongly with brain tissue-ICP in the absence of pathological obstruction to the cerebrospinal fluid flow [105].

Both feature importance (Figs 7 and 8) and SHAP summary plots (Figs 9 and 10) demonstrated that IOP_i_ was the most significant parameter in both the arterial and venous models. Moreover, there was a positive correlation between IOP_i_ and the impact on both XGB models. This indicates that ophthalmodynamometry is an important component of our image acquisition methodology. Moreover, the findings are consistent with the high correlation between retinal venous opening pressure and ICP described in ophthalmodynamometric studies in human [20–23] and animal studies [18, 19, 108–110]. Changes in the venous system from raised ICP arises from elevated downstream cerebral venous pressures and external pressure on the subarachnoid segment of the central retinal vein, which increases vascular resistance [111]. The literature has been less clear on the changes in retinal arterial circulation in this context. Moreover, it is intriguing that arterial pulsation parameters provided information sufficient to generate a prediction in spite of the less clinically recognised changes in the retinal arterial system with raised ICP. Our study demonstrated an increase in the arterial wall pulsation amplitude in the ICP_h_ group albeit at a narrower range than that of the venous system (Fig 4). In a recently published study, in addition to the retinal vascular pulse amplitude antipodal effect in response to abnormally elevated intracranial pressure, where a decrease in retinal venous pulse was accompanied by an increase in the arterial pulse proportionate to raised ICP [28]. We postulated that this effect may represent functional differences in the transmural pressure gradient between the retinal arteries and the retinal veins, where the former exhibits a higher range than the latter, therefore the retinal artery demonstrates pulsations at higher induced IOP_i_ values, this combined with raised venous pressure and reduced venous compliance and possibly augmentation of the retinal arterial pressure wave in association with elevated ICP. Other compensatory mechanisms known to modify the cerebrovascular dynamics through auto-regulatory control have been described in the retinal arterial system as well [112–115]. However, the role of these mechanisms in our observations remain unresolved. We found that the SHAP values suggested that ocular laterality was a prominent factor in the predictive model, particularly that derived from the arterial system, it could be hypothesized that hemodynamic phenomena depend on physiologically inherent asymmetry in vascular dynamics play an important role in the optimization of cardiovascular functions [116, 117]. Recent studies have demonstrated the association between ocular laterality on retinal vascular occlusions [118, 119]. Although there are anatomic reasons for retinal arterial occlusions pertaining to embolic etiologies, the reason why asymmetry plays a role in the arterial rather than the venous system remains unclear.

A major limitation of machine learning algorithms is the black-box problem, given that the nature of machine learning is based on accuracy-driven performance metrics, these models will likely continue to become even more opaque in the future, especially machine learning-generated ensembles of decision trees [120–123]. More recently, software packages like DALEX [124], breakdown [125] and XGBExplainer [126] have made some gains in terms of XGB model interpretability. A fundamental restriction inherent in all tree-based models is the inability to extrapolate target values beyond the range of the training data when making predictions, this is in contrast to linear models that can extrapolate predictions beyond the range of the training dataset [127]. Our study included a small number of participants, a larger dataset would likely offer improved generalizability. Image acquisition requires subject cooperation, which hinders the technique’s applicability in patients with cognitive or significant neurological deficits, therefore future developments would include a compact imaging system.

## Conclusion

The venous XGB model showed higher predictive accuracy compared to the arterial. Among the nine evaluated features IOP_i_ was the most important model feature in both vascular systems. Although the venous median predicted ICP showed the highest agreement with measured ICP using lumbar puncture, an independent prediction derived from the retinal arteries can provide a system of internal validation for the result. Previous studies have not considered the retinal arterial system for this purpose. The prediction model can potentially be integrated into a neurological clinical decision algorithm to evaluate the indication for lumbar puncture.

## Supporting information

S1 File (ZIP)

S2 File (ZIP)

S3 File (ZIP)
